# Tropical peanut maturation scale for harvesting seeds with superior quality

**DOI:** 10.3389/fpls.2024.1376370

**Published:** 2024-05-08

**Authors:** Gustavo Roberto Fonseca de Oliveira, Edvaldo Aparecido Amaral da Silva

**Affiliations:** Department of Crop Science, School of Agricultural Sciences, São Paulo State University (UNESP), Botucatu, Brazil

**Keywords:** *Arachis hypogaea L*., maturation profile board, optimum peanut digging, peanut seed stages, seed development

## Abstract

Determining the moment for harvesting the tropical peanut with a focus on superior seed quality is not an easy task. Particularities such as indeterminate flowering, underground fruiting and uneven maturation further increase this technical challenge. It is in this context that we aim to investigate harvest indicators based on the maturation and late maturation phases of tropical peanuts to obtain seeds with superior physiological and health quality. The plants were grown in field conditions and their development stages were carefully monitored until seed production. The water content, dry weight, germination capacity, desiccation tolerance, vigor, longevity, and seed pathogens were evaluated throughout these stages. We showed that seeds from early stages (R5 and R6) did not fully tolerate desiccation and were highly sensitive to pathogen contamination after storage (*Aspergillus*, *Penicillium*, and Bacteria). At late stages (R7, R8, and R9), the seeds had optimized vigor, longevity and bioprotection against fungi and thermal stress. The peanut maturation scale for tropical agriculture provides unique harvesting guidelines that make it possible to monitor the plants’ development stages with a focus on producing superior quality seeds.

## Introduction

The peanut is a leguminous crop native to South America, grown mostly in warm tropical environments and plays an important economic role in nations in all six continents (Stalker and Wilson, 2016; USDA, 2024). Peanut kernels are a rich source of oil, proteins and vitamins that give them the status of one of the most nutritious foods in the human diet (Arya et al., 2016; Bonku and Yu, 2020). Brazil has increased peanut cultivation by more than 100% in recent years, making it a major producer and exporter of this tropical crop (USDA, 2023; CONAB, 2024). However, there are challenges that make it difficult to increase the production efficiency of tropical peanuts, such as low physiological quality of seeds (Santos et al., 2013; Barbosa et al., 2014). Seeds with this characteristic often have poor germination efficiency (Zhou et al., 2019; Fonseca de Oliveira et al., 2022), high fungal contamination (Sudki et al., 2023), as well as difficulties in producing productive plants in the field (Song et al., 2022). For this reason, the physiological quality contributes positively to food safety (Moreno et al., 2022) and should be prioritized in order to increase efficiency in tropical peanut production.

Physiological quality is composed of germination, desiccation tolerance, vigor, and longevity, which are attributes acquired in an orderly way during seed development stages (Bewley et al., 2013). In legumes like peanuts (Okada et al., 2021), soybean (Lima et al., 2017) and cowpea (Moreno et al., 2022), the seed acquires germination capacity in the initial stages without yet tolerating desiccation. The ability to survive drastic water loss is acquired gradually in the maturation phase during seed filling (Leprince et al., 2017; Basso et al., 2018). Sequentially, the seed undergoes late maturation and acquires vigor to form seedlings in a wide range of climatic conditions (Finch-Savage and Bassel, 2016; Basso et al., 2018). In this phase, the seeds also acquire the longevity that allows them to survive longer periods of storage (Sano et al., 2016; Nadarajan et al., 2023). The fact is that for these attributes to be acquired, the seeds need sufficient time in the tropical field to complete the maturation and late maturation phases (Okada et al., 2021; Moreno et al., 2022). Thus, deciding when to harvest becomes a critical issue, because depending on the stage chosen, immature seeds with low physiological quality will be obtained (Moreno et al., 2022). Therefore, it is the timing of the harvest that will determine the success or failure of the new food production cycle that depends on the seeds produced in the previous crop season (Ebone et al., 2020; Song et al., 2022).

The issue is that there is still a gap in knowledge regarding the connection between seed physiological quality and current harvest indicators for the tropical peanut. Among the most used indicators for harvesting peanuts for food production are: i) determining the maturity of the fruits by scraping them (Williams and Drexler, 1981); and ii) identification of development stages throughout the plant cycle (Boote, 1982). In the first method, the degree of maturity of the fruits (or pods) is classified according to color (Williams and Drexler, 1981). In the second, the development stages (i.e., R1, R2, R3, R4, R5, R6, R7, R8 e R9) are described according to the morphological changes in the plant, fruits, and kernels (Boote, 1982). Both methods brought important strategies to define peanut maturity in the face of two known challenges: i) the peanut maturation process happens in the soil, and since the kernels can’t be seen, it is difficult to define the ideal harvest moment; ii) continuous flowering of the peanut plant (runner cultivars) results in fruit at multiple development stages at harvest time. These indicators work well for the production of peanuts for human consumption. However, the seeds produced in this way are not ideal for planting, because the physiological parameters essential for producing new plants are not taken into account. Considering these facts, would it be possible to use well established harvest indicators to determine the best moment for harvesting the seeds for crop production?

In the current state of knowledge, we know that the best time to harvest peanut seeds in tropical environments is the late maturation phase (Okada et al., 2021). It is during this phase that leguminous seeds acquire the molecular and chemical resources necessary for their prolonged post-harvest life (Buitink and Leprince, 2008; Leprince and Buitink, 2010; Leprince et al., 2017). The greatest challenge is to identify this phase in the field. Hypothetically, the harvest indicators created for optimized food production (Williams and Drexler, 1981; Boote, 1982) could be used together, allowing us to identify the best harvest moment to produce peanut seeds (for the planting of the next cycle) in tropical fields. Considering the technological advances this idea could bring, we investigated whether the joint use of harvest indicators for tropical peanuts would make it possible to define the moment when the seeds have superior physiological quality (late maturation phase).

## Materials and methods

### Plant material

The study was carried out with the tropical peanut crop (*Arachis hypogaea* L.). The cultivar IAC 505 (Grupo Virgínia) was used as a model, which is characterized as being high oleic, generating plants with a spreading habit and a cycle of more than 130 days. The seeds used in the study were COPERCANA (https://copercana.com.br/), located in Sertãozinho in the western region of the state of São Paulo, Brazil.

### Seed production

#### Soil and climate

The peanuts were grown at the Lageado Experimental Farm located in Botucatu, São Paulo, Brazil, in the 2021/2022 and 2022/2023 harvests in an area of 1000 m^2^. The soil was characterized as Latosol (USDA, 2015), with a history of soybean and cowpea cultivation. The area was subjected to soil preparation, which consisted of ploughing, rotary hoeing and harrowing, liming (dolomitic limestone at a dose of 2000 kg ha^-1^), furrow opening and seeding fertilizer (120 kg ha^-1^ of fertilizer formulated containing 4% N, 30% P_2_O_5_ and 10% K_2_O). The chemical attributes of the soil determined in the 0 to 20 cm layer were as follows: pH CaCl_2_: 5.69; Organic matter: 25.76 g dm^-3^; P resin: 14.07 mg dm^-3^; H+Al^3+^: 33.54 mmolc dm^-3^; K: 1.85 mmolc dm^-3^; Ca: 22.95 mmolc dm^-3^; Mg: 9.48 mmolc dm^-3^; Sum of bases: 34.27 mmolc dm^-3^; CTC: 67.82 mmolc dm^-3^; base saturation: 50.54%. Throughout the plant cycle, the variables of accumulated precipitation (1000 mm), relative air humidity (RH: 78%), maximum and minimum temperatures (28°C and 18°C, respectively) in addition to the thermal sum (1500-degree days) were monitored (**Supplementary Figure 1**) (Prela and Ribeiro, 2000; Okada et al., 2021).

#### Sowing and plant management

**Figure 1:**
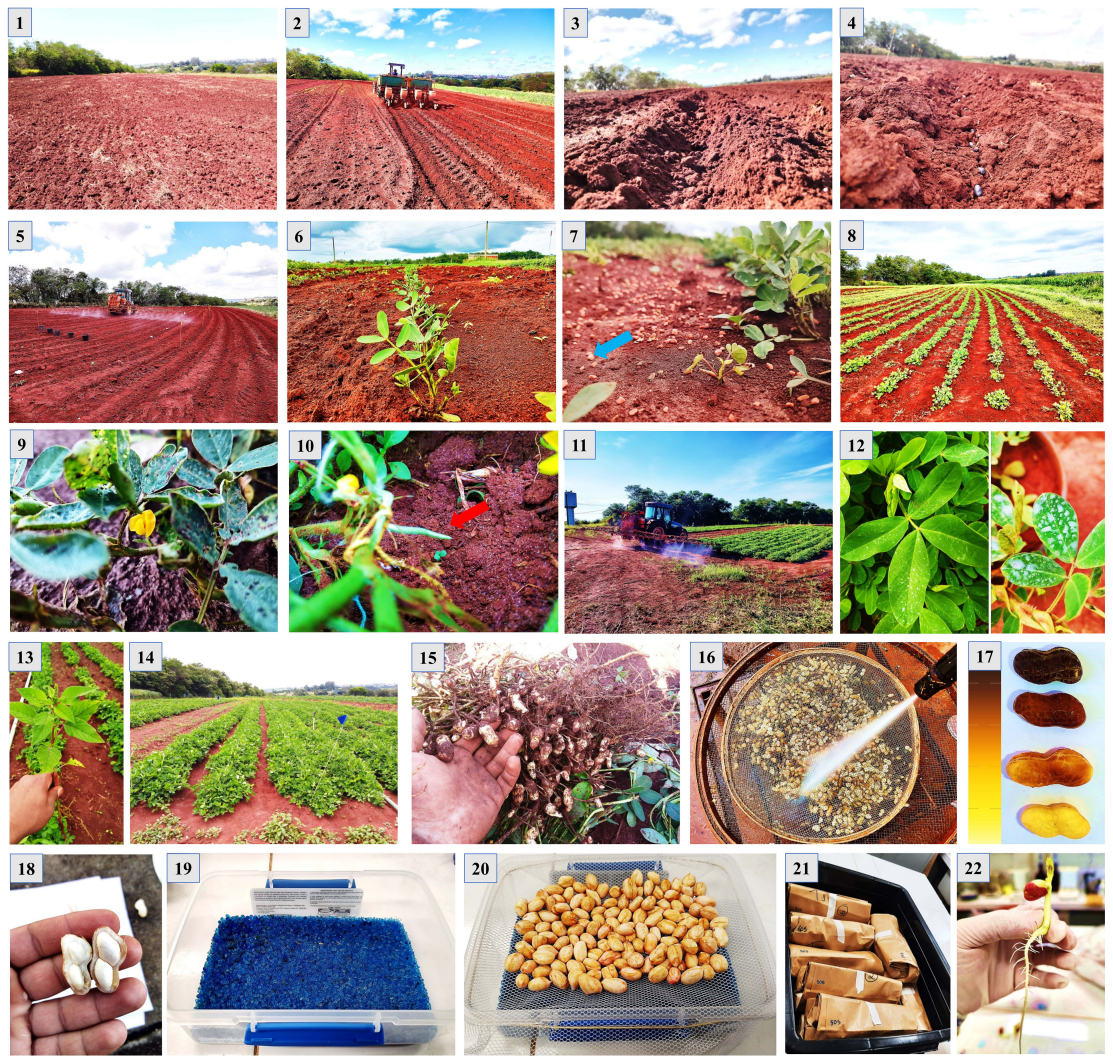
Peanut seed production under field conditions. 1) Experimental area; 2) Opening of sowing furrows; 3) Open groove; 4) Sowing seeds; 5) Application of herbicide; 6) Seedling establishment at 10 days; 7) Fertilization at 30 days; 8) Peanut plants after 30 days; 9) Beginning of flowering; 10) Emission of the gynophores (pegs); 11) and 12) Application of fungicide and insecticide; 13) Weed management; 14) Plants at 100 days, when harvesting begins; 15) Harvesting carried out manually; 16) Washing fruits in pressurized water; 17) Classification of fruits; 18) Classification of seeds; 19 and 20) Drying the seeds; 21) Seed storage; 22) Seed analysis.

Treated peanut seeds (fungicide: carboxine and thiram, 3 mL kg^-1^; insecticide: thiamethoxam, 3 mL kg^-1^) were previously inoculated (*Bradyrhizobium* sp, 600 mL ha^-1^) aiming to maximize biological nitrogen fixation. Sowing was manual and took place in November of each season (2021/2022 and 2022/2023). The seeds were distributed in the furrow equidistantly and at a depth of 5 cm with a spacing of 90 cm between rows. Overall, 23 seeds were used per meter aiming for a stand of 15 plants. After sowing, a water supplement of approximately 15 mm was established using the sprinkler method to ensure adequate seed germination. Seedling emergence occurred around 10 days after sowing. Top dressing fertilization occurred after 30 days with 50 kg ha^-1^ of K_2_O (KCl fertilizer, 60% K_2_O) supplied in parallel to the established plants. Weed, pest and disease management in the seed production field was carried out as necessary throughout the plant cycle (**Figure 1**).

#### Harvest

Peanut harvesting operations began 100 days after seedling emergence. Harvests were carried out until the number of seeds required for the planned analyses was reached (**Supplementary Table 1**). At the end of the plant cycle, of approximately 150 days, a final harvest was carried out and the proportion of maturation stages was determined for this last harvest (**Supplementary Table 2**). The plants were removed from the soil manually in the morning, always between 6 am and 8 am. The fruits were removed from the plants manually and washed in pressurized water (1500 psi). Washing took place between two concave steel sieves (60 cm in diameter) installed in opposite directions. The water jet was positioned approximately 30 cm from the fruits (between the sieves) to completely remove the exocarp and reveal the mesocarp’s color without damaging the seeds (**Figure 1**).

#### Seed development stages

**Figure 2:**
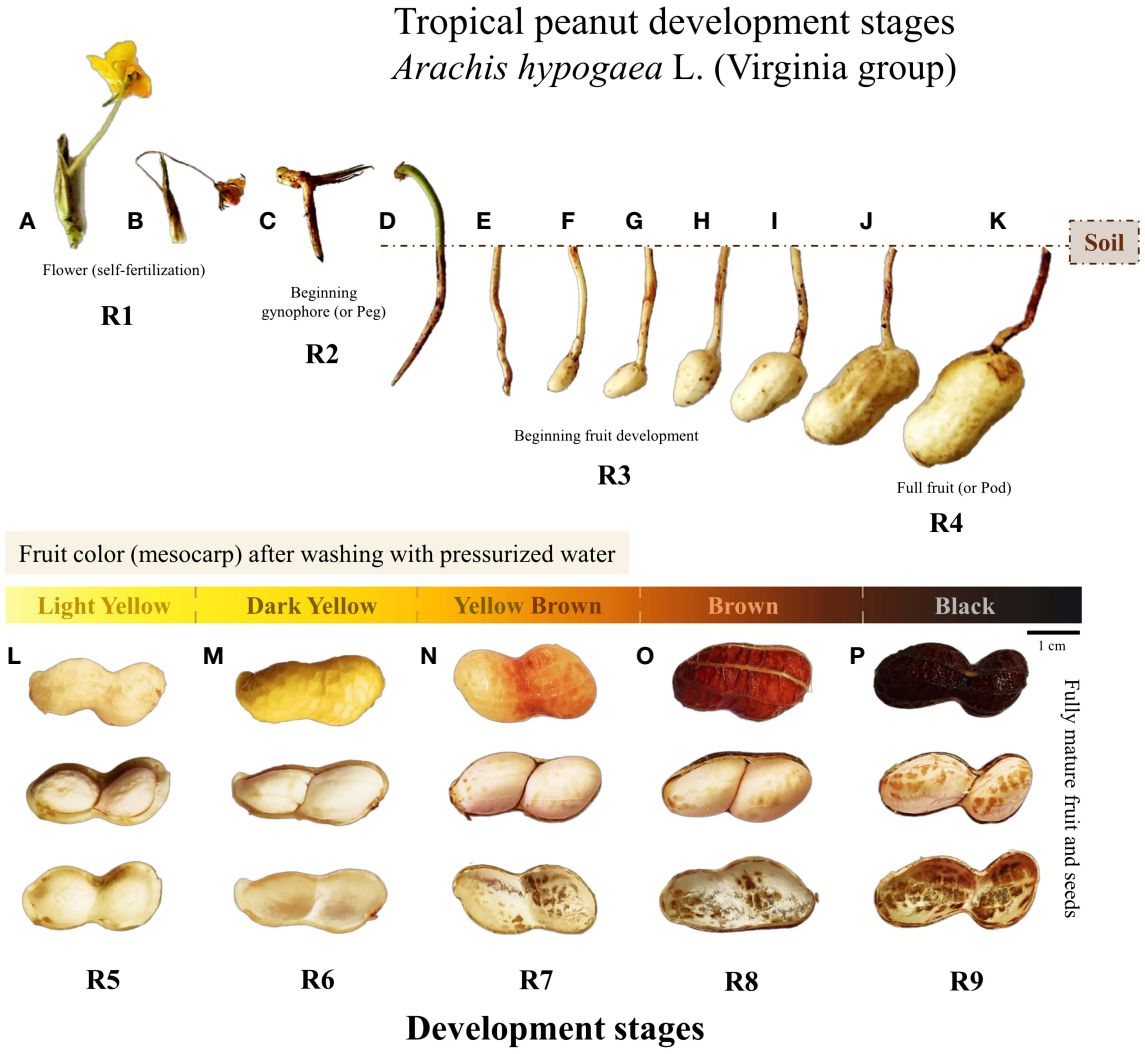
Development and morphological changes of the fruit and seed of tropical peanut (*Arachis hypogaea* L., Virginia group, cultivar IAC 505) from flowering to full maturation (Crop season 2021/2022). Descriptions in chronological order for fruit/seed maturation: **(A)** one flower opened; **(B)** flower senescence after 24 h; **(C)** development of gynophore (peg) towards the soil; **(D)** peg penetrated the soil; **(E)** peg base begins its expansion; **(F–K)** fruit development in the soil; **(L)** Light Yellow fruit color/seeds in R5 stage; **(M)** Dark Yellow fruit color/seeds in R6 stage; **(N)** Yellow Brown fruit color/seeds in R7 stage; **(O)** Brown fruit color/seeds in R8 stage; **(P)** Black fruit color/seeds in R9 stage.

Based on an adaptation of the Williams and Drexler (1981), the wet fruits were immediately sorted into five color classes: Light yellow, Dark yellow, Brown Yellow, Brown, and Black (**Figures 2L–P**). The moist, sorted fruits were then opened manually in a sterile laboratory environment. The seeds in each color class were selected using descriptions adapted from Boote (1982) for the R5, R6, R7, R8, and R9 nomenclatures. The internal visual characteristics of the fruit and seeds, such as texture, size, color, and degree of moisture, were used to complement the classification criteria (**Supplementary Table 3**). Combining the two classic peanut harvest descriptors with the commented adaptations, the classification of tropical peanut seed development stages was thus established: R5 - Light yellow; R6 - Dark yellow; R7 - Brown yellow; R8 - Brown and R9 - Black (**Figures 2L–P**). Each fruit color class corresponds to a seed development stage that represents the reproductive nomenclatures for tropical agriculture (**Figures 2L–P**).

#### Seed drying

Part of the seeds were always dried on the same day as they were harvested. Drying took place in plastic boxes (8.5 x 6.0 x 2.5 cm) containing 300g of blue silica gel (1-4mm) at the bottom. A gauze type cloth (12 cm x 10 cm) was added to the top surface of the box and secured with a rubber band. The wet seeds were added to the surface of the cloth without overlapping with each other. The lidded box was placed in a germination chamber (germinator) set at 20°C. These conditions allowed the seeds to dry quickly with a RH of around 13% inside the container. The water content was monitored every 12 h and the wet silica in the containers was changed for another drought (24h/100°C). The seeds were dried to 10% humidity (wet basis). Drying took 2 to 3 days depending on the seed’s stage of development. The dried seeds were then stored in a cold room (10°C and 55% RH) for around 90 days to carry out most of the tests. In some tests, the seeds were stored for a year. All procedures for seed production are summarized in **Figure 1**.

### Seed quality

#### Water content and dry weight

The water content of the fresh seeds and the dry weight were determined separately with five replicates of 10 seeds from each development stage (n=50). The seeds were placed in aluminum containers. For water content, the oven method was used, regulated at 105°C for 24 h (ISTA, 2020). The results were expressed as moisture content on a wet basis (%). As for dry weight, the seeds were placed in the oven set at 60°C for 72 h. The results were expressed in grams per 10 seeds.

#### Germination capacity

Germination capacity was assessed using seeds with five replicates of 25 fresh seeds from each development stage (n=125). The fresh seeds had their coat removed manually (and were immersed in a 1% hypochlorite solution for 2 minutes to sterilize them and remove fungi. The germination test took place on paper rolls moistened with deionized water at a rate of 2.5 times the weight of the dry paper. The germinator was set to 20°C at night with the light off (12 h), and 30°C during the day with the light on (12 h). Germination (radicle protrusion ≥ 2mm) was assessed 10 days after the test was set up. The procedures of removing the seed coat, treating the seeds with hypochlorite, and using alternating temperature and light (12 h night 20°C light off/12 h day 30°C light on) favored breaking the dormancy of freshly harvested peanut seeds and promoted germination with high health.

#### Desiccation tolerance

Desiccation tolerance was assessed with five replicates of 25 seeds from each stage of development (n=125) without a seed coat. The drying procedure was carried out as described under “Seed drying”. To facilitate the removal of the seed coat, the dried seeds were first distributed on a roll of paper moistened with deionized water at a rate of 2.5 times the weight of the dry paper and placed in a germinator (12 h night 20°C light off/12 h day 30°C light on). After 16 h of pre-moistening, the tegument was easily removed by hand and the seeds were sterilized and submitted to the germination test as described above. Desiccation tolerance was assessed based on the seeds’ capacity to germinate (radicle protrusion ≥ 2mm) after drying.

#### Germination and Time to 50% germination

Seed vigor based on t50 was assessed using five replicates of 25 dry seeds from each development stage (n=125). The procedures adopted for germination were described above. After 24 h of setting up the test, germination (radicle protrusion ≥ 2mm) was assessed every 4 h. The time needed to obtain 50% germination (t50) and the total germination obtained after 72 h were assessed using the Germinator software (Joosen et al., 2010).

#### Germination of aged seeds

Seed vigor based on germination after accelerated ageing was assessed with five replicates of 25 dry seeds from each stage and two further replicates of 10 seeds (n=145). Initially, the intact seeds (with the seed coat) were added to the surface of a wire mesh suspended over 40 mL of deionized water inside a hermetically sealed plastic box (11 x 1 x 3.5 cm). The boxes were placed in a chamber set at 42°C for 72 h (Rossetto et al., 2004). Two replicates of 10 seeds were used to determine the water content, as described, which was around 20% (after the aging procedure). The following procedures were then adopted: manual removal of the seed coat from dry seeds, sterilization (1% sodium hypochlorite for 2 min) and the germination conditions previously described. The germination of the aged seeds (radicle protrusion ≥ 2mm) was determined 5 days after the final test on a paper roll, as mentioned above.

#### Seedling length and dry mass

Seed vigor based on seedling length was tested using five replicates of 10 dry seeds (n=50) from each development stage. The seeds without the seed coat and treated with hypochlorite (1% for 2 min), as described above, were placed on the top third of moistened paper under the same conditions as the germination test. Two rows of 10 seeds were positioned with the hilum towards the bottom of the paper. The paper rolls were placed in transparent plastic bags sealed with rubber bands. The material was kept in a regulated germinator (12 h night 20°C light off/12 h day 30°C light on) for 7 days in an upright position. The total length of the seedling and its parts (shoot and root) was measured with a graduated ruler (cm). The dry mass of the shoot and root was assessed after 72 h in a regulated chamber at 60°C, and the results were expressed in milligrams per seedling (Krzyzanowski et al., 2020).

#### Seedling emergence, emergence speed, and normal seedlings

Seed vigor based on seedling performance in sand was carried out with five replicates of 25 (n=125) intact seeds (with the seed coat) stored for 1 year at 10°C and 55% RH (12 months of storage before the start of the test). The test was set up under field conditions (8:00 and 10:00 am) in suspended beds containing fine sand. The substrate was previously moistened with enough water for the seeds to germinate properly and kept moist when necessary. The maximum and minimum temperatures (25.7°C and 14.5°C, respectively), relative humidity (60.5%) and rainfall (14 mm) were monitored throughout the test. Seedlings with visible shoots above the substrate were counted daily and at the same time until the 21st day of installation. The seedling emergence result comprised the total number of seedlings that emerged at the end of the test. The emergence speed (index) was determined from the daily sum of the ratios between the number of seedlings that emerged and the respective reading day. Seedlings that emerged within 21 days were carefully removed from the substrate and those with perfect, unblemished, and fully developed shoot and root parts were counted as normal seedlings (Krzyzanowski et al., 2020).

#### Establishment of plants in the field

Seed vigor, based on plant establishment in the field, was assessed using five replicates of 20 intact seeds (with the seed coat) (n=100) previously stored for 1 year at 10°C and 55% RH (12 months of storage before the start of the test). Except for the liming operation, the same experimental area where the seeds were produced was subjected to the soil preparation and sowing procedures described above (item “Seed production”). For each seed development stage, five parallel 1-meter rows were formed, 90 cm apart. The seeds were used without any type of chemical treatment. Immediately after sowing, the area was subjected to water supplementation of around 15 mm by sprinkling to promote seed germination and seedling establishment. Established plants were counted 30 days after sowing using the presence of the first fully expanded leaves as a criterion. Weed control was carried out manually throughout the experiment. Maximum and minimum temperatures (36.4°C and 17.3°C, respectively), relative humidity (RH: 71.1%) and rainfall (72.6 mm) were monitored. The results were expressed as a percentage of plants established in the field.

#### Longevity and germination of stored seeds

The longevity of the seeds based on their viability during storage was determined using 420 dry intact seeds (with the seed coat) (n=420) from each development stage. The seeds were placed on a gauze type cloth (12 cm x 10 cm) attached with rubber bands to the top surface of a plastic box (8.5 x 6.0 x 2.5 cm) containing saturated salt solution (NaCl) at the bottom. The hermetically sealed box was placed in a germinator at 35°C and 75% RH. Five replicates of 10 seeds were sampled at seven intervals between 14 and 20 days. At each sampling, two replicates of 5 seeds were used to determine the water content, which was around 7% to 8% throughout the test. The procedures for removing the seed coat and sterilizing the seeds described above were carried out. Germination took place on paper rolls under the conditions already described and viability was assessed (radicle protrusion ≥ 2 mm) at 10 days. Longevity was defined as the time in days that the seeds (R5, R6, R7, R8, and R9) lost 50% of their viability (p50) during storage. The vigor of the seeds stored for 71 days was assessed using the germination percentage (radicle protrusion ≥ 2 mm) of the seeds at 10 days.

#### Seed health quality

The sanitary quality of the seeds was assessed using the filter paper method (Henning, 2017; ISTA, 2020). Seven replicates of 10 dry, intact seeds without any type of treatment (n=70) and previously stored for 1 year at 10°C and 55% RH (storage before the start of the test) were used. The seeds were placed on filter paper (diameter 10 cm) that had been previously sterilized (autoclave at 120°C for 20 min) and moistened with water (deionized and autoclaved) at a rate of 2.5 times the weight of the dry paper. Three papers were added to sterilized Petri dishes (70% alcohol). The seeds were added to two moistened papers and covered with a third. The plates were then sealed with parafilm. The material was placed in a germinator set to 20°C with constant light (white fluorescent) for 7 days. The pathogens *Aspergillus* ssp, *Penicillium* ssp and Bacteria (*Bacillus* sp.) were identified under a stereoscopic microscope and counted per seed unit. In the same test, germination (radicle protrusion ≥ 2mm) was assessed. The results were expressed as a percentage.

## Statistical Design

### Analysis of variance

The data was analyzed using a completely randomized design with five development stages (R5, R6, R7, R8, and R9) as the variation factor, with five repetitions (n=25). The data was subjected to ANOVA after checking the assumptions of normality and homoscedasticity using the Shapiro-Wilk and Bartlett tests. The averages were compared using the Tukey’s test with a significance level of 5% (**Supplementary Table 5**). The longevity data (p50) was obtained from the generalized linear model with the Cauchy function at 5% significance level. R software was used for the analyses (R Core Team, 2024).

### Principal components analysis

The data observed for the seed physiological and health quality variables presented so far were subjected to principal components analysis (PCA). Furthermore, Permutational Multivariate Analysis of Variance - PERMANOVA One-way (Canoco 5 software) was used to test the similarity of data behavior (Bray-Curtis similarity index) in two groups. The groups were: i) early seed stages (R5 and R6); and ii) late seed stages (R7, R8 and R9). The significance of the groups was tested at a level of 1% (Anderson, 2017).

### Random Forest and correlation analysis

The Random Forest, a machine learning algorithm (Brieuc et al., 2018), was used to model the relationship between seed development stages and 22 seed quality variables. When applied to predicting seed development stages, the Mean Decrease Gini (MDG) method quantifies the extent to which each predictor variable contributes to the accuracy of the model. Variables with higher MDG values are considered more important in predicting seed development stages. Correlation analysis was calculated using the Spearman method. R software was used for the analyses (R Core Team, 2024).

## Peanut maturation scale

Original images of peanuts immediately after washing in pressurized water were used to create maturation scales. Descriptions of the external (mesocarp) and internal (endocarp) parts of the fruit were made for each stage of development (R5, R6, R7, R8, and R9). Original images of open fruits containing two seeds each (characteristic of the Virginia group) were also used. The seed quality scale, represented by an ascending line from 0 to 90%, was obtained considering the most important variable detected in the Random Forest analysis to differentiate the development stages of tropical peanuts. The tropical peanut maturation scale for seed production was also prepared in the Portuguese and Spanish languages, available in the Supplementary Material (**Supplementary Figures 2**, **3**). Finally, practical suggestions for using the information in the tropical agricultural context were prepared (**Supplementary Table 4**).

## Results

### Water content and seed filling

**Figure 3:**
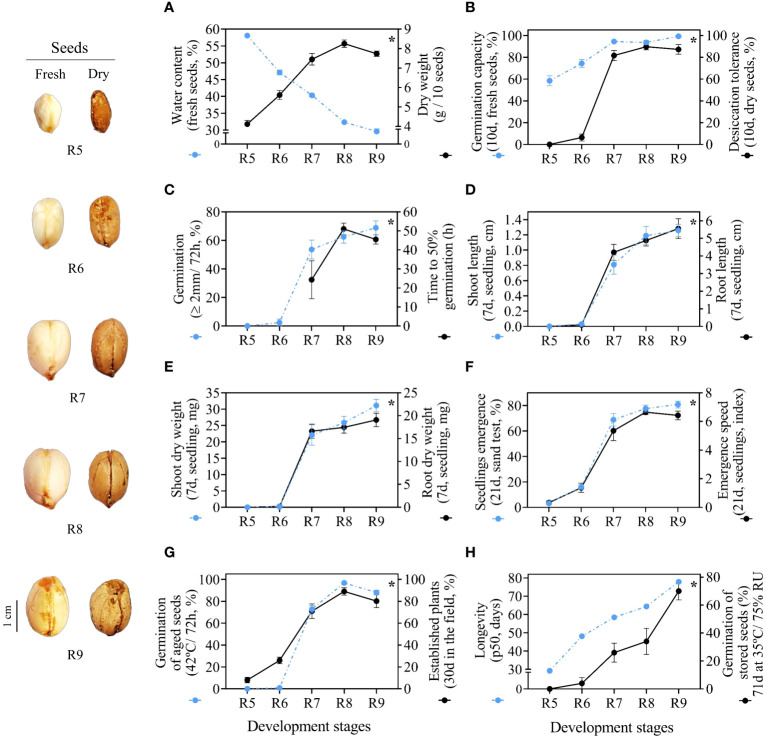
Physiological quality of seeds. Crop season 2021/2022: **(A)** water content and dry weight; **(B)** germination capacity and desiccation tolerance; **(C)** radicle protrusion and time to 50% germination; **(D)** length of shoot and root of seedlings; **(E)** dry weight of shoot and root of seedlings; **(F)** seedling emergence and seedling emergence speed. Crop season 2022/2023: **(G)** radicle protrusion of aged seeds (at 41°C/72 h) and established plants; **(H)** seed longevity and radicle protrusion. All results contain the standard deviation of the average and a minimum significance level (*) by the F Test (*p value* ≤ 0.05).

The notable morphological changes of the fruits in the process of maturation (**Figures 2L–P**) occur in parallel with the physical changes of the seeds (**Figure 3A**). As regards water content, a high degree of seed moisture was observed at the beginning of development for stages R5 (58%) and R6 (47%) (**Supplementary Table 5**). From stage R7 the water content of 40% rapidly decreased to 32% in stage R8 and again to 30% in stage R9. The water dynamics of the seeds were inverse to the accumulation of dry weight (**Figure 3A**), which increased significantly until the R7 stage (~7g/10 seeds). This point defined the maturity of the seed mass or the maximum filling of reserves. In general, seeds from late stages (R7, R8 and R9) presented different water contents and similar and stable dry weight (**Figure 3A**, **Supplementary Table 5**).

### Germination capacity and desiccation tolerance

Germination of fresh seeds occurred in the initial stages R5 and R6 with a high degree of humidity. However, seeds at the R5 stage were highly sensitive to water loss and did not germinate after drying. Interestingly, even with low germination (6.4%), the first signs of desiccation tolerance were only observed in seeds at the R6 stage. Desiccation tolerance was fully acquired at the R7 stage with germination at around 90%. In the following stages, R8 and R9, the results remained stable (**Figure 3B**). In general, late-stage seeds tolerate desiccation and do not lose viability after the drying step.

### Time to 50% Germination

At the beginning of the development process, seeds from the initial stages (R5 and R6) did not germinate (**Figure 3C**). This did not result in t50 values, which was notable evidence of low seed vigor. It was interesting to note that by the time desiccation tolerance was fully acquired at the R7 stage (**Figure 3B**), t50 began to be accounted for (**Figure 3C**). Overall, it took about 50 h for half of the seeds to fully germinate at the R8 and R9 stages. In relation to total germination, seed performance increased significantly between development stages (**Supplementary Table 5**). The R9 stage seeds had maximum germination in a short evaluation period (72 h) (**Figure 3C**).

### Seedling length and Dry weight

The few seeds that germinated at the R6 stage gave rise to seedlings with poorly developed shoots and roots over the 7 days of evaluation (**Figure 3D**). Seeds from the late stages (R7, R8 and R9) produced seedlings with larger roots (~4 cm and 5 cm) and higher dry mass accumulation in the shoot (~16 mg and 19 mg) and root (~22 mg and 31 mg) (**Supplementary Table 5**). Seeds at the R8 stage were able to produce seedlings with larger shoots, similar to those found in the R9 stage (around 1.2 cm). In general, seeds from late stages were more efficient in transferring reserves to seedlings, which was evidenced by well-developed shoots and roots (**Figures 3D, E**).

### Seedling emergence and emergence speed

Under uncontrolled environmental conditions, seeds from the early stages (R5 and R6) occasionally gave rise to seedlings with reduced emergence capacity (3% and 12%, respectively). Notably, there was a higher percentage of seedlings that emerged at the R7 stage (~60%) and the results remained statistically similar at the R8 and R9 stages (~74% and ~80%, respectively). It was interesting to note that only seeds from stages R7, R8 and R9 showed higher emergence speed (**Figure 3F**). This constitutes a characteristic that expresses the superior potential of late-stage seeds to rapidly establish seedlings under broad environmental conditions (**Figure 3F**).

### Aged seeds and established plants in the field

Seeds up to the R6 stage aged at a high temperature (42°C for 72 h) germinated very little (0.8%). Germination increased considerably in the following stages, reaching 72.8% in the R7 stage and 98.8% in the R8 stage, which had a maximum performance similar to the R9 stage (**Figure 3G**). After 1 year of storage (10°C/55% RH), seeds at early stages (R5 and R6) gave rise to approximately 8% to 26% plants after 40 days in the field (**Supplementary Table 5**). The results were notably higher in the R7, R8 and R9 stages, where the percentage of established plants was around 70 to 89% (**Figure 3G**). This means that seeds from late stages were better able to guarantee the initial formation of a new peanut production cycle even after prolonged storage (one year) (**Figure 3G**).

### Seed longevity

Interestingly, only R9 stage seeds had the highest germination (70%) after 71 days stored under conditions of heat stress and high humidity (35°C/75% RH). Physiologically, maximum seed quality was acquired at the R9 stage (**Figure 3H**). In general, this ability to germinate even under long exposure under these conditions increased as the seeds entered late maturation from the R7 stage onwards (**Supplementary Table 5**). Seeds from late stages (R7, R8 and R9) took longer to lose 50% viability (p50) during storage. This means they had superior longevity (**Figure 3H**). In the context of peanut maturation, seeds at stages R7, R8 and R9 have superior storage potential and post-harvest vigor (**Figure 3H**).

### Seedling performance before and after storage

**Figure 4:**
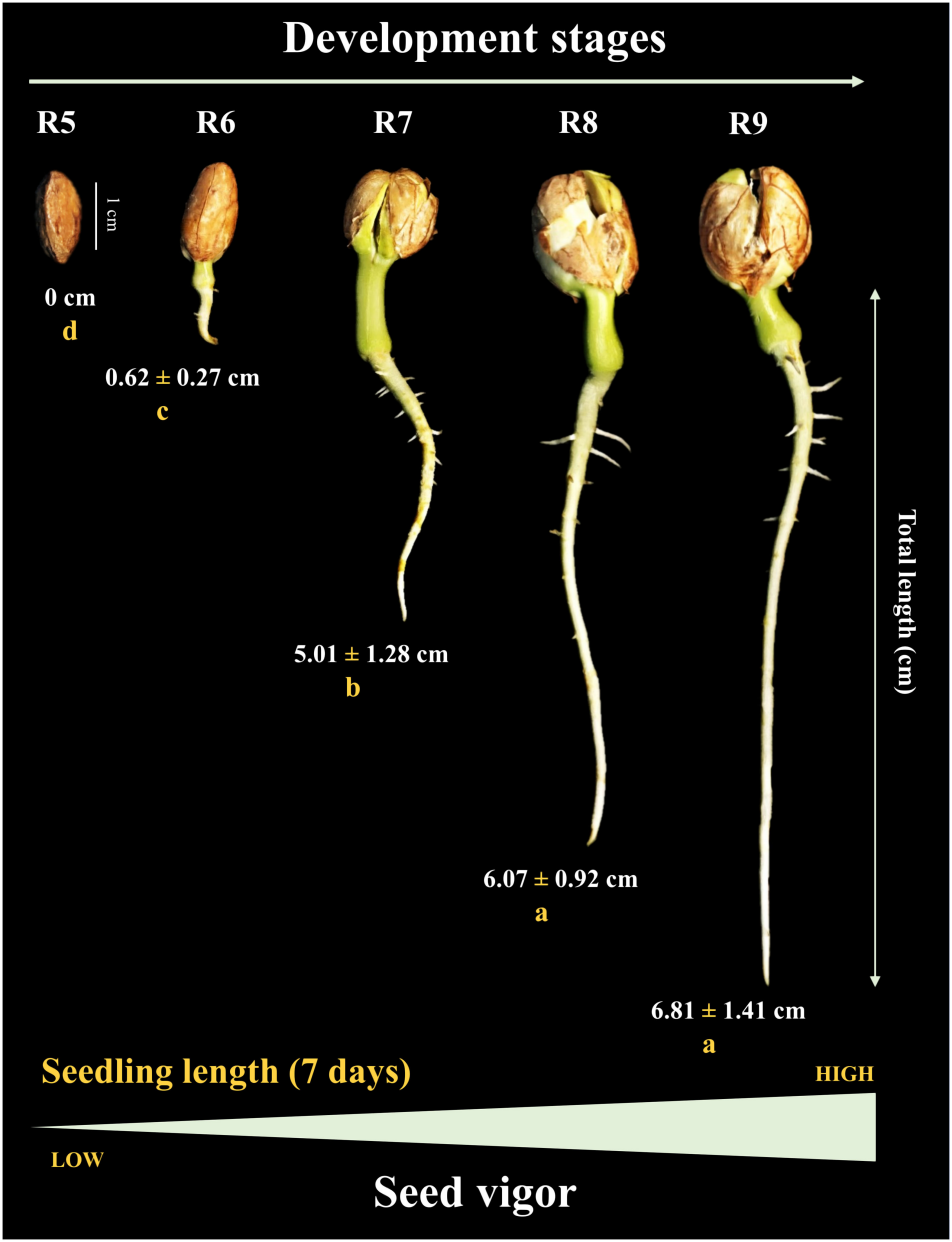
Seedling length at 7 days of age as a function of seed development stages (Crop season 2021/2022). All results contain the standard deviation of the average. Different lowercase letters indicate a significant difference between the averages by Tukey test (*p value* ≤ 0.05).

**Figure 5:**
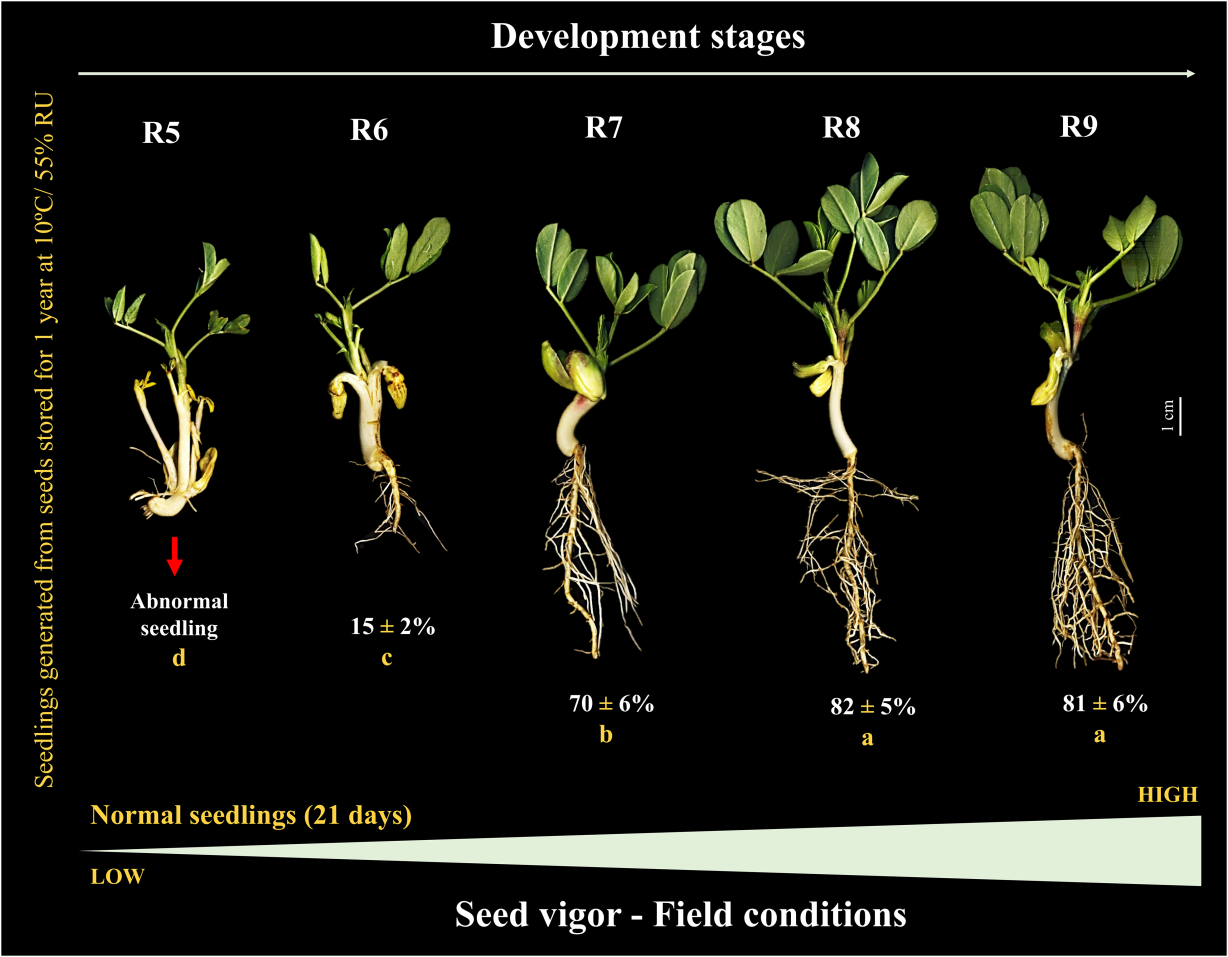
Peanut seedlings from seeds at different stages of their development stored for 1 year at 10°C and 55% RH (Crop season 2022/2023). Seedlings obtained under field conditions 21 days after sowing. All results contain the standard deviation of the average and a minimum significance level by the F Test (*p value* ≤ 0.05). Different lowercase letters indicate a significant difference between the averages by Tukey test (*p value* ≤ 0.05).

Under controlled conditions (temperature and humidity) prior to storage, the seeds generated seedlings, during 7 days of evaluation, only from the R6 stage (< 1 cm) onwards. The total length of the seedlings increased notably at the R7 stage (~5 cm). Seeds from the R8 stage gave rise to seedlings with a maximum size (~6 cm) similar to the R9 stage (**Figure 4**). Interestingly, after 1 year of storage (10°C/55 RH), the seedling size result followed the same behavior as the percentage of normal seedlings (without defects or damage) in uncontrolled field conditions. For example, although some seeds at the R5 stage eventually germinated, they were not considered normal (absence of a developed root). The formation of normal seedlings was 15% at the R6 stage and 70% at the R7 stage. Notably, the maximum percentage of normal seedlings (80%) was obtained for seeds at the R8 stage, similar to R9 (**Figure 5**). This means that the seeds acquired maximum vigor for the establishment of seedlings under broad environmental conditions at the R8 stage, and that this vigor did not change after storage.

### Seed health

**Figure 6:**
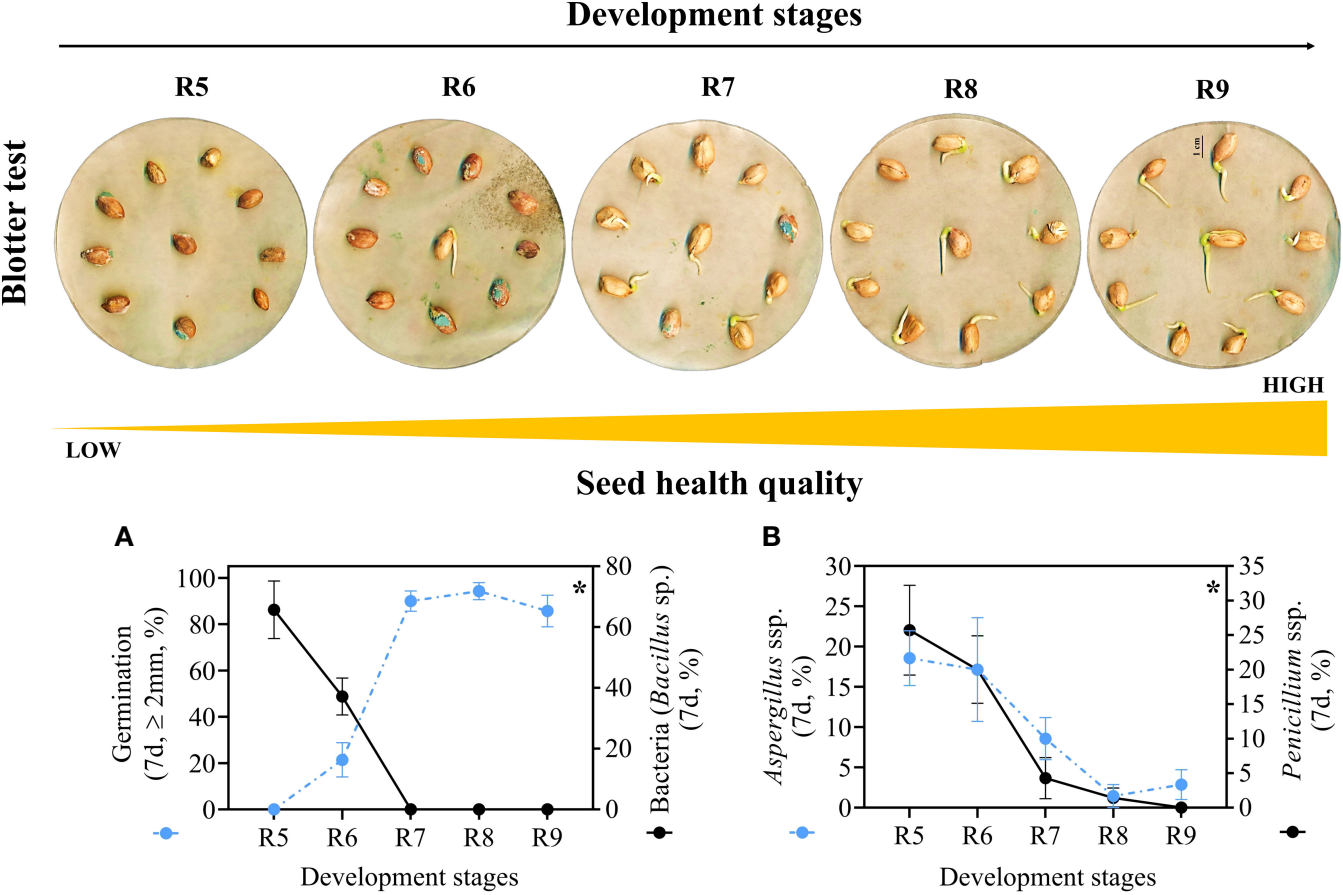
Health quality of seeds from different development stages (Crop season 2022/2023). **(A)** Radicle protrusion of seeds obtained in the blotter test and percentage of seed contamination by bacteria (*Bacillus* sp.). **(B)** percentage of *Aspergillus* ssp. and *Penicillium* ssp. in the seeds. All results contain the standard deviation of the average and a minimum significance level (*) of 5% by the F Test (*p value* ≤ 0.05).

**Figure 7:**
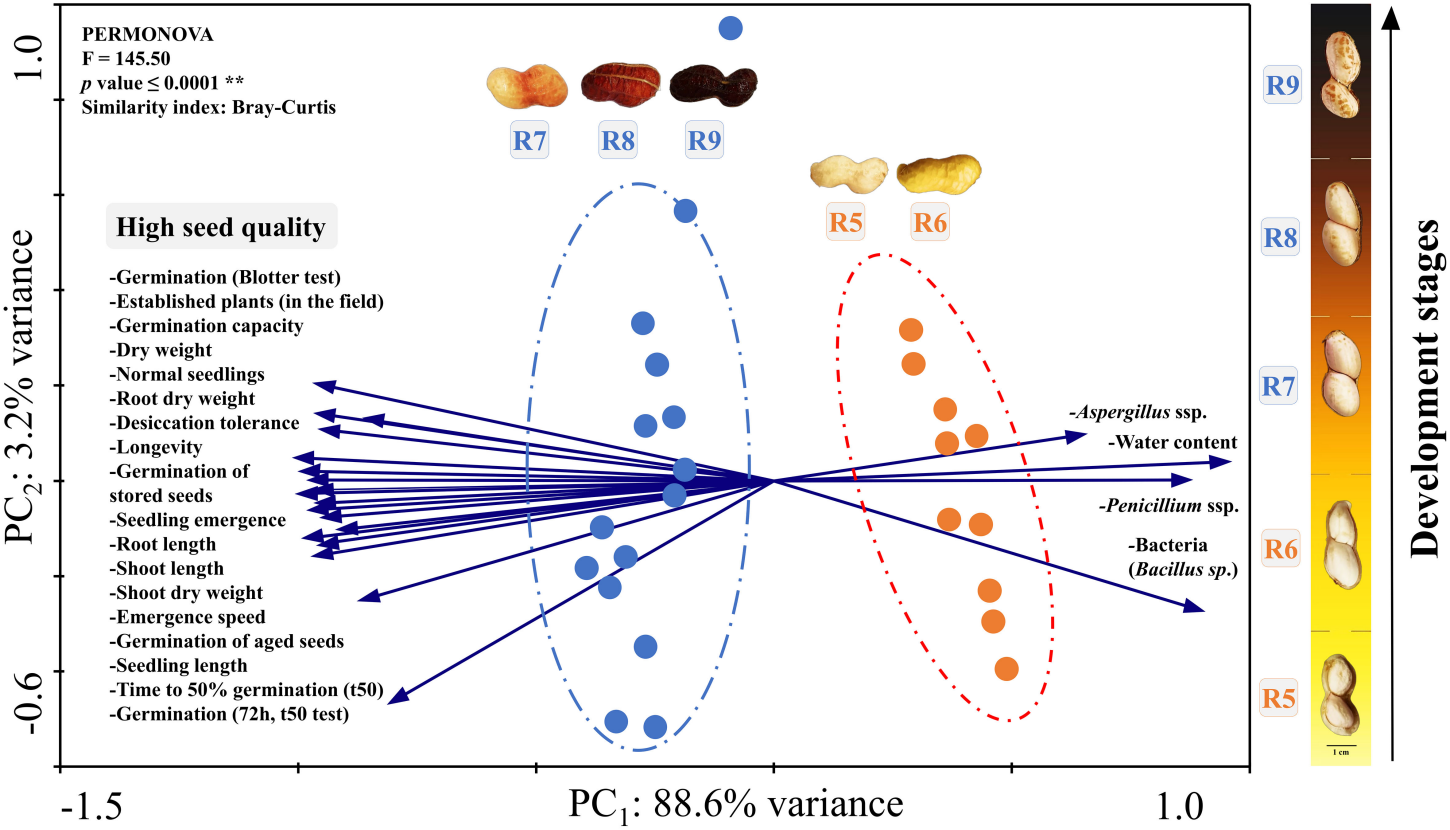
Principal component analysis (PCA). All the seed quality variables evaluated (Crop Seasons 2021/2022 and 2022/2023) were plotted considering two groups: early seed stages (R5 and R6) and late seed stages (R7, R8 and R9). The circles represent these two groups which were significantly different by the PERMANOVA test according to the Bray-Curtis similarity index (*p* value ≤ 0.0001^**^).

The presence of pathogens decreased as the seeds entered the late maturation phase from the R7 stage onwards (**Figure 6**). Seeds at stages R5 and R6 showed reduced germination (0 and 21.4%, respectively) and a high percentage of bacteria (65.7% and 37.1%, respectively). Regarding fungi: i) in the R5 stage, around 18% and 25% of *Aspergillus* ssp and *Penicillium* ssp were observed, respectively; and ii) in the R6 stage, this respective percentage was around 17% and 20% (**Figure 7**). Interestingly, in the late stages there was no presence of bacteria and seed germination was around 85% to 90%, although they were in an environment contaminated with pathogens (**Supplementary Table 5**). Furthermore, the seeds showed low susceptibility to *Aspergillus* ssp (~1% to 3%) and *Penicillium* ssp (<2%) (**Supplementary Table 5**). In general, seeds from stages R7, R8 and R9 showed superior resilience to the proliferation of pathogenic microorganisms (**Figure 6**).

### Principal components analysis

The results of the entire research were divided into two distinct groups (PERMANOVA, p value < 0.001) by the similarity index (Bray-Curtis) and with 88.6% of variance in PCA_1_ and 3.2% in PCA_2_ (**Figure 7**). In the first group, the highest values of germination, desiccation tolerance, vigor and seed longevity were strongly related to the late stages (R7, R8 and R9). On the other hand, the second group consists of the initial seed stages (R5 and R6). The main characteristics of these stages were: i) data associated with high contamination by *Aspergillus, Penicillium* and bacteria (*Bacillus* sp.); ii) high water content (**Figure 7**) in the case of seeds before drying (**Figure 3A**); and iii) none of the related physiological quality parameters. In general, through the PCA, it was evident that the R7, R8 and R9 stages should be prioritized in monitoring the peanut harvest aiming for superior seed quality.

### Random forest and correlation analysis

**Figure 8:**
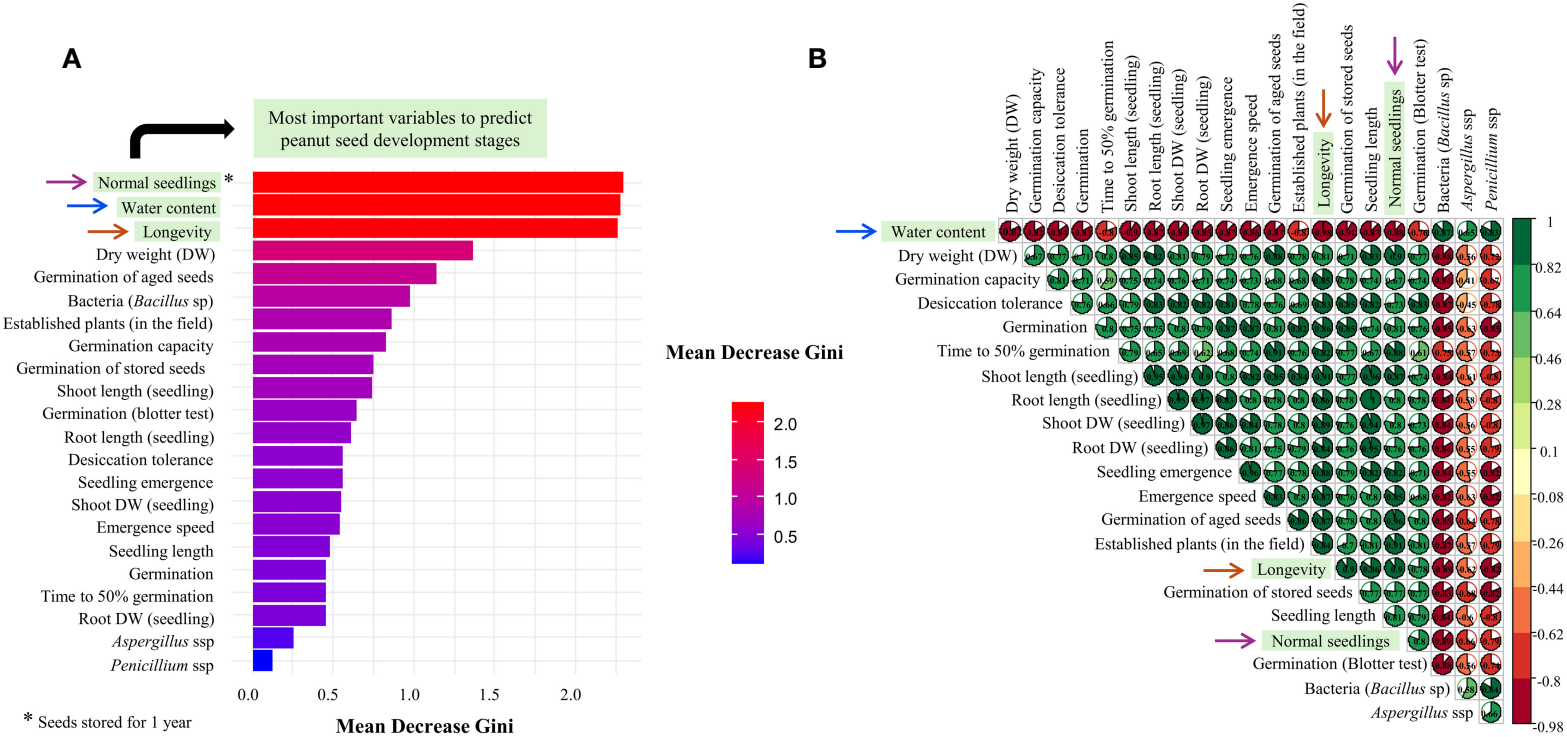
Mean Decrease Gini for each seed quality variable in the Random Forest analysis to discriminate peanut seeds at different development stages: R5, R6, R7, R8, and R9 **(A)**. Correlation matrix – Spearman method **(B)**. The arrows highlighted in both figures indicate the most important variables in the maturation of tropical peanut seeds.

The ranking of the observed data, using the Random Forest tool, made it possible to detect in order of importance the best variables to discriminate the development stages of peanut seeds. The most important variables were: 1°) Normal Seedlings; 2°) Water content; and 3°) Longevity (**Figure 8A**). For the first highlighted variable: i) the percentage of normal seedlings was positively correlated with dry weight (r= 0.9) and with the physiological quality attributes of the seed (0.67 ≥ r ≤ 0.91); and ii) the correlation was negative for contamination by fungi and bacteria (pathogens). Subsequently, except in the case of pathogens (0.65 ≥ r ≤ 0.87), the higher the water content of the seed, the lower its mass, germination capacity, desiccation tolerance, vigor, and longevity (-0.76 ≤ r ≥ -0.98). Finally, interestingly, superior seed longevity was negatively correlated with water content (r < -0.98) and pathogen contamination (-0.62 ≤ r ≥ -0.89). In general, longevity was directly proportional to the physiological quality of the seeds, which enabled normal seedling production months after initial storage (0.78 ≥ r ≤ 0.9) (**Figure 8B**).

### Peanut maturation scale

**Figure 9:**
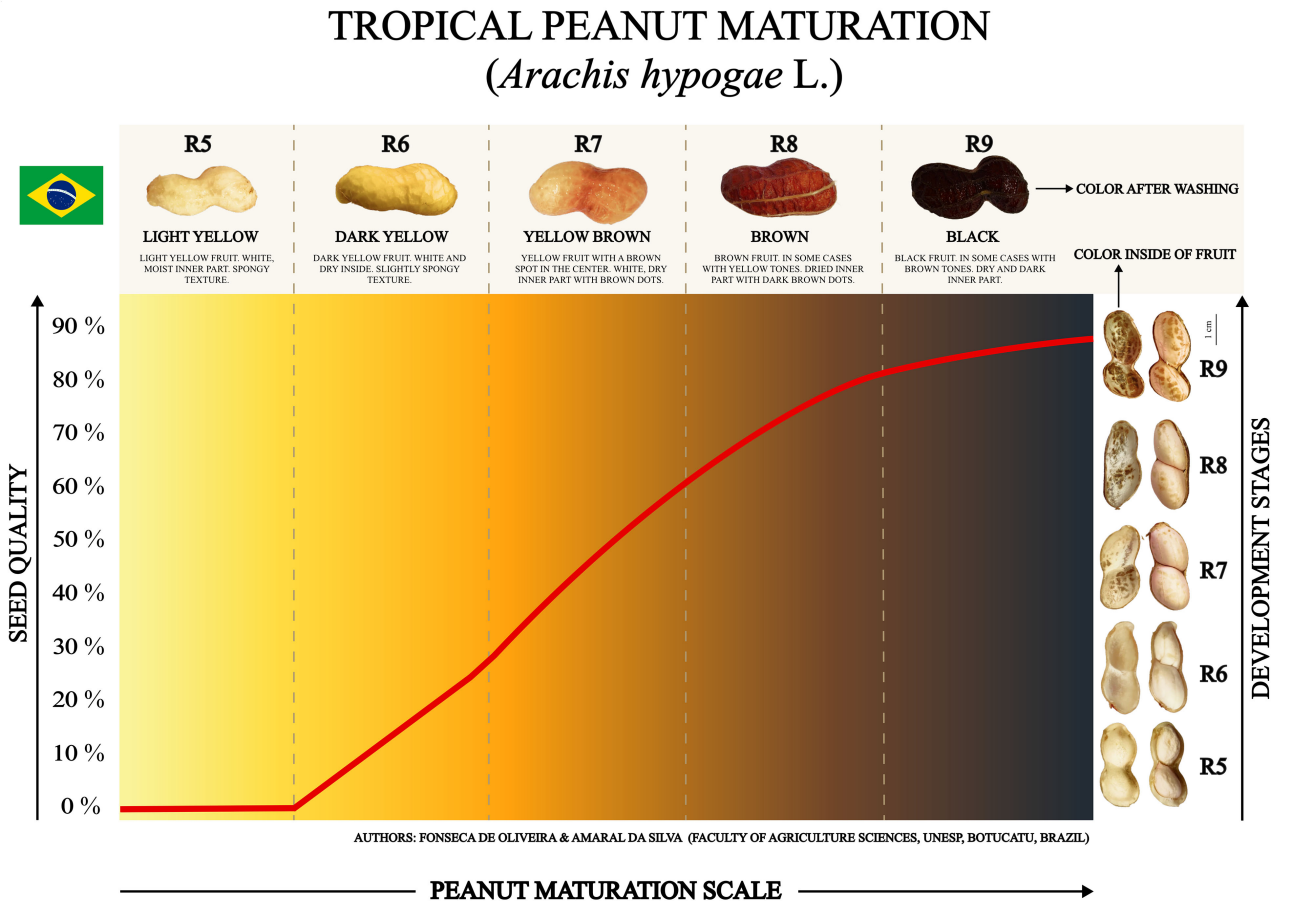
Peanut maturation scale for harvesting seeds with superior quality. Seed quality as a function of development stages identified through morphological changes in fruit color and seed aspects. The red line indicates the acquisition of quality of tropical peanut seeds represented by the variable “Normal Seedlings” obtained with seeds stored for 1 year. The fruit and seed colors are original.

The tropical peanut maturity scale summarized all research results (**Figure 9**). Seed quality was expressed based on the most important variable for classifying the stages of seed development obtained in the Random Forest analysis (machine learning): Normal seedlings (**Figures 5**, **8A**). The information allows monitoring the potential of newly harvested peanut seeds to form seedlings in the field after the storage phase. Thus, each stage of development classified at the time of harvest is related to a future percentage of seed quality. This way, it is possible to understand what the seed’s likely performance will be in the following crop season (new production cycle). For example: at stage R5 the seeds do not have the potential to generate plants in the field, therefore, they have 0% physiological quality; at stage R6 the seeds present around 20% quality; in stage R7, around 70% quality; in stages R8 and R9 around 90% quality (**Figure 9**).

## Discussion

### Water dynamics and seed filling

Water dynamics govern physical and biochemical changes in legume seeds (Righetti et al., 2015; Lima et al., 2017; Okada et al., 2021). Changes in water content occur in parallel with cellular events during seed development (Aldana et al., 1972; Bewley et al., 2013). For example, cell division and expansion in the embryogenesis phase depend on high humidity for the synthesis of nucleic acids and the formation of seed tissues (Setter and Flannigan, 2001; Armenta-Medina et al., 2021). The moisture required for this process after fertilization (stages R1 to R4) reflects the higher seed water content in the following development stages (**Figure 2**). In the beginning of the filling phase (stage R5), the flow of assimilates from the plant to seeds occurs under high water demand (Copeland and McDonald, 2001; Okada et al., 2021). This means that water contents of around 60 to 40% are critical until the R7 stage (**Figure 3A**) for the accumulation of reserves such as sugars, oils, and proteins (Pattee et al., 1974). These reserves are crucial for cellular protection in face of sudden variations of embryonic humidity that occur until the end of late maturation (Sattler et al., 2004; Chatelain et al., 2012; Lima et al., 2017). Stability in reserve accumulation is achieved at water contents of around 40 to 30% in the late stages (**Figures 2**, **3**). Water dynamics can help identify at which reproductive stage most seeds in field are (**Figure 8**). Therefore, with this information it is possible to harvest more mature seeds with improved chemical composition.

### Surviving the dry state

The ability to survive desiccation (losing more than 90% of water) is a characteristic of angiosperm seeds and other organisms (Gaff and Oliver, 2013; Oliver et al., 2020). The natural desiccation of peanut seeds in the soil and subsequent artificial drying to 10% humidity (**Figures 3A, B**) cause structural changes in the cellular membrane system (Hoekstra et al., 2001). Part of the reserves accumulated in the filling phase play an essential protective role when under these circumstances (Smolikova et al., 2021). Among the main seed reserves we have: i) LEA proteins (Chatelain et al., 2012); ii) HSP proteins (Wehmeyer and Vierling, 2000); and iii) sugars from the raffinose family (Hell et al., 2019). A system involving these compounds preserves the protein structure and imparts molecular stability as water is removed from the developing seed (**Figures 3A, B**) (Buitink and Leprince, 2004). This system allows the late-stage seeds to remain viable after desiccation and to germinate when rehydrated (**Figure 3B**) (Verdier et al., 2013; Lima et al., 2017). This means that ending the peanut cycle with a high proportion of immature stages (i.e., R5 and R6) can cause seed death during the drying step (**Figure 3B**). The solution to optimizing the acquisition of desiccation tolerance is to establish a judicious harvest based on development stages (Lima et al., 2017; Moreno et al., 2022). This increases the chances of obtaining peanut seeds with superior viability.

### Late maturation: extending the shelf life of dry seeds

In late maturation, when the reserves are practically stabilized, the seeds complete the acquisition of vigor and longevity under low levels of humidity (Basso et al., 2018; Okada et al., 2021). The “long life” mechanisms of leguminous seeds that preserve their biodiversity are then established (**Figure 3H**) (Verdier et al., 2013; Righetti et al., 2015). In particular, the formation of the vitreous cytoplasm, the action of antioxidants and the repair mechanisms in cells, ensure the metabolic stability of dry seeds during storage (Buitink and Leprince, 2004, 2008; Zinsmeister et al., 2020). These molecular and chemical defenses (i.e., tocopherols) delay cellular aging after desiccation (Finch-Savage and Bassel, 2016; Ramtekey et al., 2022). Interestingly, the protective capacity described for acquiring longevity is exclusive to late stages (**Figure 3H**) (Lima et al., 2017; Zhou et al., 2019). In them, vigor is preserved as much as possible so that the seeds germinate and produce seedlings months after the harvest (**Figures 4**, **5**) (Sano et al., 2016; Reed et al., 2022). However, harvesting seeds with these benefits requires assiduous monitoring of late maturation (Leprince et al., 2017; Basso et al., 2018). It is in this context that the peanut harvest indicators linked to the development stages presented have great practical utility (**Figure 2**) (Williams and Drexler, 1981; Boote, 1982). Through the joint utilization of these well-established harvest indicators, late maturation can be accurately monitored in tropical fields enabling the obtention of peanut seeds with maximum physiological quality.

### Seed maturity: bioprotection against pathogens

Seed maturity determines its bioprotection (**Figure 6**) (Commey et al., 2021; Mendu et al., 2022). Seeds accumulate phenolic compounds, flavonoids, carotenoids, and other antioxidant agents as they develop (Wan et al., 2016; Zinsmeister et al., 2020). In addition, they acquire a thick cell wall to protect against biotic stresses both physically and chemically (Polo et al., 2020; Ninkuu et al., 2023). All these defense agents create a natural barrier against pathogen infection, especially *Aspergillus* ssp, a fungus of global concern in peanuts (**Figure 6B**) (Kumar et al., 2017; Miura et al., 2019; Norlia et al., 2019; Commey et al., 2021). Taking this into account, it is understandable that seeds from the more immature stages still have deficient protection, as they have not completed their development (**Figure 6**) (Smýkal et al., 2014; Okada et al., 2021). In the late stages, most of the immune response configurations are molecularly programmed (Lima et al., 2017; Nayak et al., 2017). Thus, the biological meaning of seed maturity boils down to having resources to survive in the dry state, protect itself for long periods, germinate and provide new plant cycles in time and space (Sano et al., 2016; Song et al., 2022). In tropical agriculture, bioprotection benefits can be obtained with the harvesting guidelines proposed here (**Supplementary Tables 3**, **4**).

### Maturation scale for harvesting seeds resilient to tropical stresses

Harvesting seeds that are resilient to environmental adversities is a highly relevant strategy given the numerous stresses that crops are subjected to in the field (Sun et al., 2019; Reed et al., 2022). Peanut seeds go through post-harvest stages that are very prone to oxidative stress (Barbosa et al., 2014; Groot, 2022). This stress can be mitigated with seed maturity (**Figures 5**, **6**). In tropical peanut-producing nations, examples such stresses are: i) drying in the field exposed to the weather; ii) low availability of cold storage structures; and iii) sowing under extreme variations in temperature and soil moisture (Nakagawa and Rosolem, 2011; Sun et al., 2019; USDA, 2023). Only seeds from late stages (high seed quality) are able to tolerate biotic and abiotic stresses with maximum efficiency (**Figure 7**) (Sano et al., 2016; Ellis, 2019; Zinsmeister et al., 2020). This is because a significant part of the mechanisms that mitigate the deterioration of seeds in the dry state are dependent on the moment they were harvested (Leprince et al., 2017; Ramtekey et al., 2022). Therefore, the maturation scale, when assisting in harvest programming, is an opportunity for technological advancement in the peanut production sector in different countries (**Figure 9**) (USDA, 2023).

## Conclusion and perspectives

Peanut harvest indicators based on development stages make it possible to define the best time to obtain seeds with superior quality in tropical fields. This information has the potential to help farmers with two main technical challenges: i) if the harvest occurs too early, most of the seeds will be in the initial stages and with reduced physiological and health quality; and ii) if the harvest is carried out too late, the gynophores (pegs) that provide physical support to the fruits will have less mechanical resistance and will be lost in the soil along with the desired seeds. It is in the midst of this paradox of harvesting too early or too late that the maturation scale has great value in tropical agriculture. It contributes to the efficient monitoring of peanut late stages so the harvest can occur at the most opportune moment, that is, when seeds are less susceptible to pathogens and have the highest physiological quality. It is logical to think that any practice that provides benefits to seed quality has a direct connection with crop performance in subsequent harvests. At the very least, the proposed harvesting guidelines represent a technological step towards increasing peanut production efficiency in tropical nations.

## Data availability statement

The original contributions presented in the study are included in the article/**Supplementary Material**. Further inquiries can be directed to the corresponding author.

## Author contributions

GF: Writing – review & editing, Writing – original draft, Visualization, Software, Methodology, Investigation, Formal analysis, Data curation, Conceptualization. EA: Writing – review & editing, Visualization, Validation, Supervision, Project administration, Conceptualization.

## Supplementary material

Supplementary Figure 1 Environmental conditions during seed production (crop seasons 2021/2022 and 2022/2023).

Supplementary Figure 2 Tropical peanut maturation (*Arachis hypogaea* L.) in Portuguese language.

Supplementary Figure 3 Tropical peanut maturation (*Arachis hypogaea* L.) in Spanish language.
