# Supplementary figures and images for: Tropical peanut maturation scale for harvesting seeds with superior quality
Source: Front Plant Sci. 2024 May 8;15:1376370. doi: 10.3389/fpls.2024.1376370 (PMC11113016; doi:10.3389/fpls.2024.1376370)

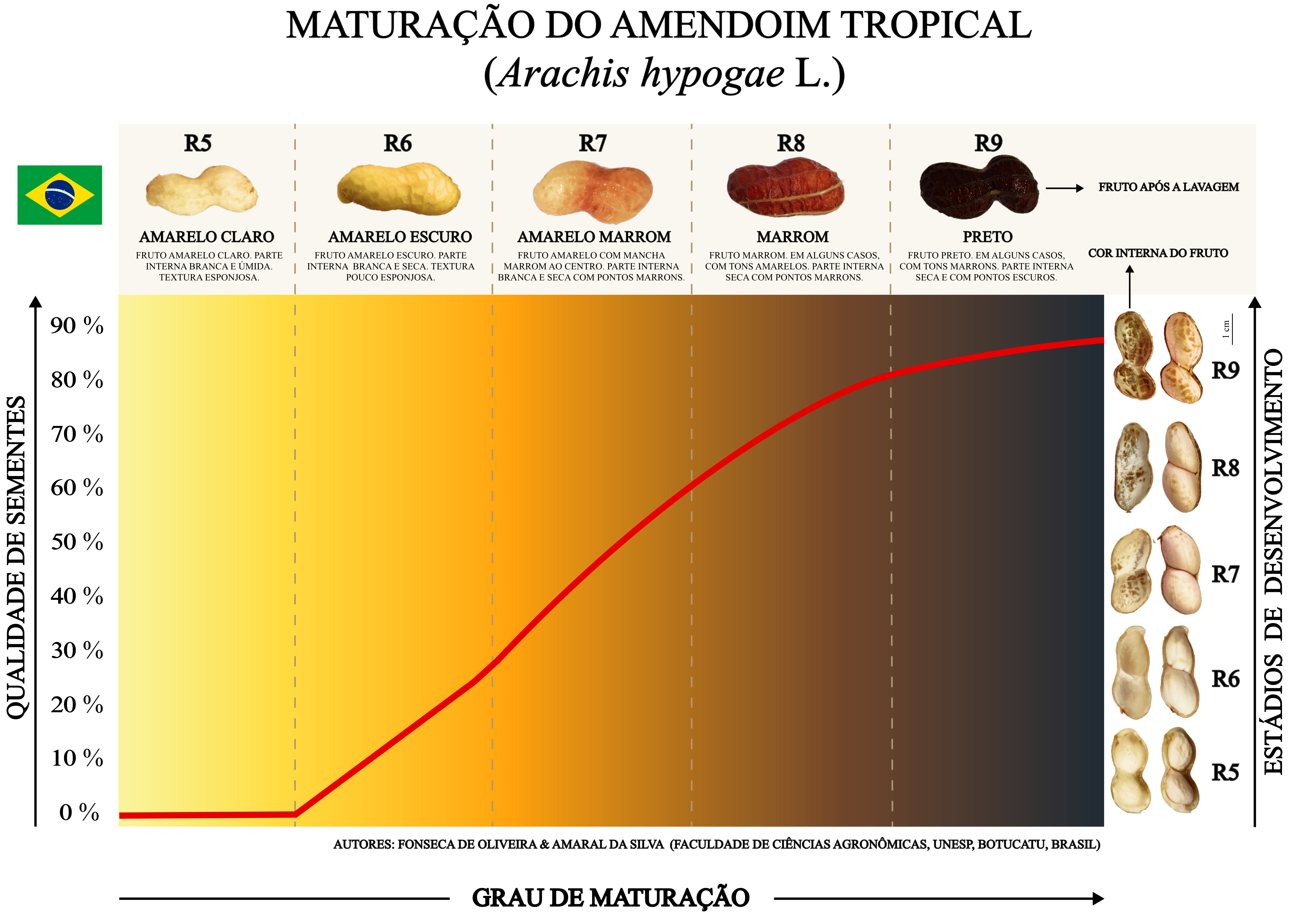

Supplement: Supplementary Figure 2 — Tropical peanut maturation (Arachis hypogaea L.) in Portuguese language. [file Image_2.jpeg]

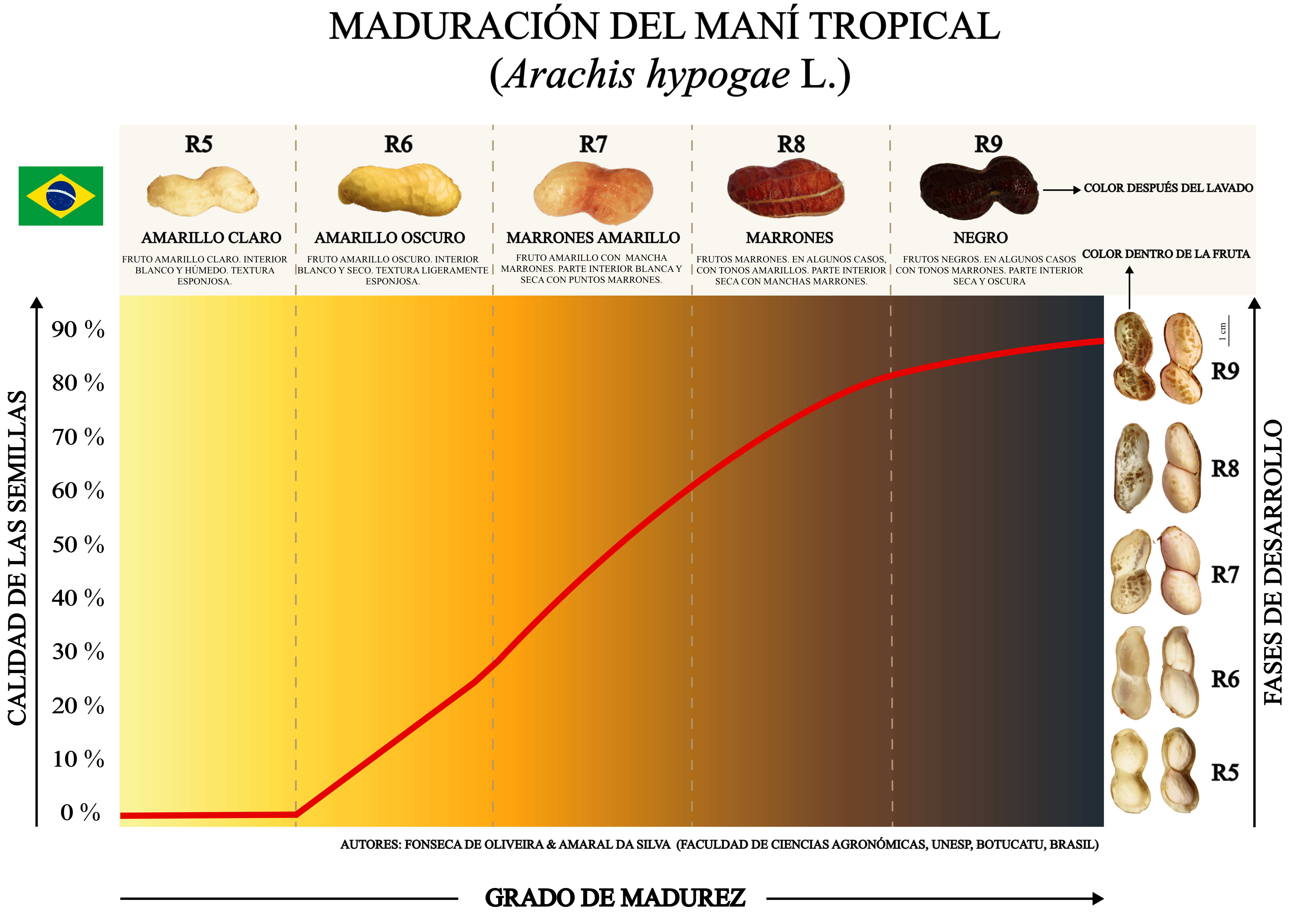

Supplement: Supplementary Figure 3 — Tropical peanut maturation (Arachis hypogaea L.) in Spanish language. [file Image_3.jpeg]
