# Supplementary material for: Tropical peanut maturation scale for harvesting seeds with superior quality
Source: Front Plant Sci. 2024 May 8;15:1376370. doi: 10.3389/fpls.2024.1376370 (PMC11113016; doi:10.3389/fpls.2024.1376370)
Supplement: Supplementary file 4 [file Table_1.docx]

**Supplementary Table 1.** Details regarding the 22 variables measured, number of seeds, and method used to evaluate peanut seed health, water content, dry weight, germination capacity, desiccation tolerance, vigor, and longevity during development stages.

| Variables | Number of seeds  (per seed stage) | | | Methods |
| --- | --- | --- | --- | --- |
| 1- Water content | | 50 | (ISTA, 2020) * | |
| 2- Dry weight | | 50 |  |  |
| 3- Germination capacity | | 125 |  |  |
| 4- Desiccation tolerance | | 125 |  |  |
| 5- Time to 50% germination (t50)  6- Germination t50 | | 125 | (Joosen et al., 2010) * | |
| 7- Germination of aged seeds | | 145 | (Rossetto et al., 2004) * | |
| 8- Seedling length  9- Shoot length of seedling  10- Root length of seedling  11- Shoot dry weight of seedling  12- Root dry mass of seedling | | 50 | (Nakagawa, 1994) * | |
| 13- Seedling emergence  14- Emergence speed of seedling  15- Normal seedlings | | 125 | (Krzyzanowski et al., 2020) * | |
| 16- Established plants in the field | |  | (Krzyzanowski et al., 2020) * | |
| 17- Longevity  18- Germination of storage seeds | | 420 | (Okada et al., 2021) * | |
| 19- *Aspergillus* ssp  20- *Penicillium* ssp  21- Bacteria (*Bacillus* sp)  22- Germination (Blotter test) | | 70 | (Henning, 2015)  (ISTA, 2020)  * | |
| Total seeds used (22 variables/ seed stage) | | 1285 | Information considered before setting up the experiment | |
| Additional seeds (safety margin) | | 215 |  |  |
| Total seeds by developmental stage | | 1500 | Seeds produced in the field | |

* Variables obtained using adaptations presented in this article.
