# Supplementary material for: Tropical peanut maturation scale for harvesting seeds with superior quality
Source: Front Plant Sci. 2024 May 8;15:1376370. doi: 10.3389/fpls.2024.1376370 (PMC11113016; doi:10.3389/fpls.2024.1376370)
Supplement: Supplementary file 5 [file Table_2.docx]

**Supplementary Table 2.** Proportion of fruit/seed development stages considered to define the optimal harvesting point for peanut plants in the field (crop season 2021/2022 and 2022/2023). The plants (cultivar IAC 505) were collected with around 70% of maturation (around 70% of fruit at stages R7, R8, and R9).

| **Repetitions of 200 fruits ^a^** | **Seed development stages (proportion%)** | | | | | | |
| --- | --- | --- | --- | --- | --- | --- | --- |
|  | **R5** | **R6** | **R7** | | **R8** | **R9** | |
| **Crop season 2021/2022** | | | | | | | |
| 1 | 4 | 18 | 20 | | 39 | 20 | |
| 2 | 4 | 13 | 31 | | 35 | 18 | |
| 3 | 4 | 18 | 32 | | 29 | 16 | |
| 4 | 4 | 21 | 28 | | 32 | 15 | |
| 5 | 2 | 19 | 20 | | 39 | 20 | |
| Average | 3.5 | 17.6 | 26.3 | | 34.8 | 17.8 | |
| Sum | 21.1 | | 78.9 | | | | |
|  |  | |  | | | | |
| **Crop season 2022/2023** | | | | | | | |
| 1 | 5 | 26 | 26 | 37 | | | 5 |
| 2 | 3 | 25 | 26 | 40 | | | 5 |
| 3 | 4 | 27 | 25 | 38 | | | 6 |
| 4 | 6 | 20 | 30 | 40 | | | 5 |
| 5 | 5 | 31 | 27 | 29 | | | 9 |
| Average | 4.6 | 25.8 | 26.8 | 36.8 | | | 6 |
| Sum | 30.5 | | 69.6 | | | | |

**^a^** Peanut fruit obtained from randomly harvested plants in the experimental area (field) around 150 days after crop emergence. Only well-formed fruits were considered when determining the harvest point.
