# Supplementary material for: Tropical peanut maturation scale for harvesting seeds with superior quality
Source: Front Plant Sci. 2024 May 8;15:1376370. doi: 10.3389/fpls.2024.1376370 (PMC11113016; doi:10.3389/fpls.2024.1376370)
Supplement: Supplementary file 6 [file Table_3.docx]

**Supplementary Table 3.** Criteria used to classify the maturation stages of tropical peanuts (*Arachis hypogaea* L). Fruit characteristics after washing in pressurised water, wet seeds, and seed water content. Descriptors used to classify the five stages of peanut seed development (cultivar IAC 505), crop seasons 2021/2022 and 2022/2023 (Lageado Experimental Farm, Botucatu, São Paulo, Brazil). The letter "X" indicates the use of the described characteristics of the fruit and/or seed to classify the development stages.

| **Stages** | **Beginning changes** | **Descriptions of the first stages of peanut development in tropical fields** | | | | | |
| --- | --- | --- | --- | --- | --- | --- | --- |
| **R1** | Flower | A single flower blooming at one of the plant's nodes | | | | | |
| **R2** | Gynophore (peg) | A single elongated gynophore visible on the plant directed towards the soil, but not yet penetrating it | | | | | |
| **R3** | Fruit development | The gynophore has penetrated the soil, and its extremity (ovary) is dilated | | | | | |
| **R4** | Full fruit (or pod) | A fully expanded fruit with cultivar-specific dimensions and a completely white mesocarp | | | | | |
|  |  | |  |  | | | |
|  | **Fruit characteristics after washing** | | **Characteristics of fresh seed (immediately after harvesting)** | **Characteristics used to classify seed development stages *** | | | |
|  |  |  |  | **Water content %**  **2021/2022** | **Water content %**  **2022/2023** | **Seeds** | **Fruits** |
| **R5** | - Light yellow fruit mesocarp  - White internal color  - Internal appearance spongy and completely moist to the touch | | - Completely white seed  - Two seeds touch  - Seeds do not fill the fruit cavity  - Moist to the touch | 58.07 ± 0.5 | 56.90 ± 0.9 | X | X |
| **R6** | - Dark yellow fruit mesocarp  - Internal color of the shell completely white  - Internal appearance spongy and predominantly moist to the touch | | - Completely white seed  - Seeds fill predominantly the fruit cavity  - Seeds moist to the touch | 47.12 ± 0.8 | 47.45 ± 1.5 | X | X |
| **R7** | - Yellow mesocarp with a brown spot in the centre  - Interior of the shell predominantly white with brown spots  - Internal aspect of the shell moist to the touch | | - Predominantly white color with brown tones  - Seeds fill the fruit cavity  - Start of dark spots on the seed coat  - Seeds less moist to the touch | 40.32 ± 0.4 | 39.47 ± 1.4 | X | X |
| **R8** | - Brown mesocarp  - Inside of shell partly white with brown spots  - Internal aspect of the shell predominantly dry | | - Predominantly brown seeds  - Dark spots on the seed coat  - Predominantly dry to the touch | 32.34 ± 0.3 | 32.30 ± 1.0 | X | X |
| **R9** | **-** Black mesocarp  - Inside of the shell predominantly brown with distinct dark spots  - Internal aspect of the fruit rough and dry | | - Completely brown seed  - Clear dark spots on the seed coat  - Predominantly dry to the touch | 29.83 ± 0.6 | 30.69 ± 0.7 | X | X |

***** Visual aspects of fruit and seeds classified based on the color of the washed fruit, the appearance of the fresh seed and confirmed by determining the water content of the fresh seed. Harvesting began around 120 days and ended around 140 days after plant emergence.
