# Supplementary material for: Tropical peanut maturation scale for harvesting seeds with superior quality
Source: Front Plant Sci. 2024 May 8;15:1376370. doi: 10.3389/fpls.2024.1376370 (PMC11113016; doi:10.3389/fpls.2024.1376370)
Supplement: Supplementary file 7 [file Table_4.docx]

**Supplementary Table 4.** Suggestions for practical use of the tropical peanut maturation scale.

| Procedures | Descriptions |
| --- | --- |
| **Standard classification** | |
| Sampling plants in the field | Weekly collection of two repetitions of five peanut plants with fruit at different (random) points in the production field as of 100 days after plant emergence. |
| Fruits | Removal of the fruit from the plants and selection of 200 intact, seeded, and well-formed fruits (without signs of rot or pest attack) for each repetition. Fruits in the early stages of formation should not be considered. |
| Washing | Washing the fruit in pressurised water (1500 psi). The fruits can be held between sieves or on a metal support (e.g. made of wire mesh) that makes it possible to contain the fruits during washing and allows the mesocarp to be removed by wet scarification. |
| Classification of fruit after washing | The sample of 200 wet fruits should be selected immediately after washing. An alternative is to leave the washed fruit immersed in water. In this case, sorting can be done a few hours after washing. Sort the 200 fruits into stages R5 - Light yellow; R6 - Dark yellow; R7 - Brown yellow; R8 - Brown and R9 - Black. The white coloured fruits are not included in the classification as they are not fully formed. |
| Results | Calculate the proportion (%) of each stage of development for the two replicates of 200 fruit, based on the visual criteria of the fruit presented in the tropical peanut maturation scale. |
| Harvest decision - seed field | Harvest the peanut plants when the average value of the samples is at least 70% of the fruits in stages R7 - Yellow brown; R8 - Brown and R9 - Black. This means that most of the seeds in the peanut field have acquired high quality (superior physiological and health quality). |
| **Alternative classification** | |
| Sampling plants in the field | Collection of two replicates of five peanut plants with fruit at different (random) points in the production field as of 100 days after plant emergence. |
| Fruits | Removal of the fruit from the plants and selection of 100 intact, seeded, and well-formed fruits (without signs of rot or pest attack) for each repetition. Fruits in the early stages of formation should not be considered. |
| Classification | Manually open the wet fruit and classify the stages of development based on the visual characteristics of the seed and fruit, such as: internal colour of the fruit and aspects of the seed, such as color, texture, moisture, size, and filling inside the fruit cavity. |
| Results | Calculate the proportion (%) of stages R7, R8 and R9 for the two repetitions of 100 fruits. |
| Harvest decision - seed field | Harvest the peanut plants when the average value of the samples is 70% of the fruits in stages R7 - Yellow brown; R8 - Brown and R9 - Black. This means that most of the seeds in the peanut field have acquired high quality (superior physiological and health quality). |
