# Supplementary material for: Tropical peanut maturation scale for harvesting seeds with superior quality
Source: Front Plant Sci. 2024 May 8;15:1376370. doi: 10.3389/fpls.2024.1376370 (PMC11113016; doi:10.3389/fpls.2024.1376370)
Supplement: Supplementary file 8 [file Table_5.docx]

**Supplementary Table 5.** Statistical data. Peanut seed quality during development stages.

| Seed stages | Water  content ^1^  % | Dry  weight  g/ 10 seeds | Germination capacity  % | Desiccation tolerance  % | t50  Radicle ≥ 2mm  hours | Germination  25ºC, 72h, % | Root length  seedling  cm |
| --- | --- | --- | --- | --- | --- | --- | --- |
| R5 | 58.07 ± 0.5 a | 4.12 ± 0.2 c | 58.4 ± 4.6 c | 0.0 ± 0.0 b | - | 0.0 ± 0.0 d | 0.0 ± 0.0 b |
| R6 | 47.12 ± 0.8 b | 5.61 ± 0.2 b | 74.4 ± 3.5 b | 6.4 ± 3.2 b | - | 2.4 ± 2.4 cd | 0.1 ± 0.03 b |
| R7 | 40.32 ± 0.4 c | 7.45 ± 0.3 a | 94.4 ± 1.6 a | 81.6 ± 4.6 a | 24.3 ± 10.0 b | 53.6 ± 6.4 bc | 4.2 ± 0.12 a |
| R8 | 32.34 ± 0.3 d | 8.25 ± 0.2 a | 93.6 ± 3.4 a | 89.6 ± 2.7 a | 51.0 ± 5.8 a | 62.4 ± 4.3 b | 4.9 ± 0.12 a |
| R9 | 29.83 ± 0.6 e | 7.73 ± 0.1 a | 99.2 ± 0.8 a | 87.2 ± 4.4 a | 45.5 ± 2.4 a | 68.8 ± 4.9 a | 5.6 ± 0.08 a |
|  | Shoot length seedling  cm | Shoot dry weight  mg | Root dry weight  mg | Seedling emergence  % | Emergence speed  index | Germination  of aged seeds  41ºC, 72h, % | Established plants  Field conditions % |
| R5 | 0.0 ± 0.0 c | 0.00 ± 0.0 c | 0.00 ± 0.0 b | 3.2 ± 0.8 b | 0.33 ± 0.1 c | 0.0 ± 0.0 d | 8 ± 2.5 b |
| R6 | 0.1 ± 0.09 c | 0.15 ± 0.14 c | 0.18 ± 0.18 b | 12.0 ± 3.3 b | 0.99 ± 0.4 c | 0.8 ± 0.8 d | 26 ± 2.9 b |
| R7 | 0.8 ± 0.45 b | 22.1 ± 3.1 b | 16.06 ± 1.5 a | 60.0 ± 11.1 a | 4.52 ± 0.9 b | 72.8 ± 3.4 c | 71 ± 6.5 a |
| R8 | 1.2 ± 0.31 a | 25.9 ± 1.9 ab | 17.45 ± 1.3 a | 74.4 ± 3.7 a | 6.51 ± 0.2 ab | 96.8 ± 0.8 a | 89 ± 3.1 a |
| R9 | 1.3 ± 0.55 a | 31.1 ± 1.9 a | 19.06 ± 1.5 a | 80.8 ± 2.6 a | 6.64 ± 0.2 a | 88.8 ± 2.1 b | 80 ± 5.8 a |
|  | Germination of stored seeds  35ºC, 75% RU  % | Seedling length  cm | Normal Seedlings  after storage 10ºC, 55% RU  % | Seed health quality (after storage: 10ºC, 55% RU) | | | |
|  |  |  |  | Germination  ≥ 2mm  % | Bacteria (*Bacillus* sp)  % | *Aspergillus* ssp  % | *Penicillium* ssp  % |
| R5 | 0.0 ± 0.0 d | 0.00 ± 0.00 d | 0.0 ± 0.0 d | 0.0 ± 0.0 c | 65.7 ± 9.4 a | 18.57 ± 3.4 a | 25.71 ± 6.4 a |
| R6 | 4.0 ± 4.0 cd | 0.62 ± 0.27 c | 15.0 ± 2.0 c | 21.4 ± 7.3 b | 37.1 ± 6.0 b | 17.14 ± 6.4 a | 20.00 ± 4.8 a |
| R7 | 26.0 ± 6.7 bc | 5.01 ± 1.28 b | 70.0 ± 6.0 b | 90.0 ± 4.3 a | 0.0 ± 0.0 c | 8.57 ± 2.6 b | 4.28 ± 2.9 b |
| R8 | 34.0 ± 9.2 b | 6.07 ± 0.92 a | 82.0 ± 5.0 a | 94.3 ± 3.6 a | 0.0 ± 0.0 c | 1.57 ± 1.4 b | 1.42 ± 1.4 b |
| R9 | 70.0 ± 6.3 a | 6.81 ± 1.41 a | 81.0 ± 6.0 a | 85.7 ± 6.8 a | 0.0 ± 0.0 c | 2.86 ± 1.8 b | 0.0 ± 0.0 b |

^1^ The averages were compared by the Tukey test after checking the significance of 1% by the F-test. Different lowercase letters in the same column indicate a significant difference between the averages (Tukey test; *p* ≤ *value* 0.05). All averages were presented with the respective standard deviation (±).
